# Sub-second and multi-second dopamine dynamics underlie variability in human time perception

**DOI:** 10.1101/2024.02.09.24302276

**Published:** 2024-02-09

**Authors:** Renata Sadibolova, Emily K. DiMarco, Angela Jiang, Benjamin Maas, Stephen B. Tatter, Adrian Laxton, Kenneth T. Kishida, Devin B. Terhune

**Affiliations:** 1 Department of Psychology, Goldsmiths, University of London; London SE14 6NW, UK.; 2 Department of Psychology, Institute of Psychiatry, Psychology and Neuroscience, King’s College London; London SE5 8AB, UK.; 3 School of Psychology, University of Roehampton; London SW15 4JD, UK.; 4 Neuroscience Graduate Program, Wake Forest School of Medicine; Winston-Salem, NC, 27157, USA.; 5 Department of Translational Neuroscience, Wake Forest School of Medicine; Winston-Salem, NC, 27157, USA.; 6 Virginia Tech – Wake Forest University School of Biomedical Engineering and Sciences, Wake Forest School of Medicine; Winston-Salem, NC, 27157, USA.; 7 Department of Biomedical Engineering, Wake Forest School of Medicine; Winston-Salem, NC, 27157, USA.; 8 Department of Neurosurgery, Wake Forest School of Medicine; Winston-Salem, NC, 27157, USA.

## Abstract

Timing behaviour and the perception of time are fundamental to cognitive and emotional processes in humans. In non-human model organisms, the neuromodulator dopamine has been associated with variations in timing behaviour, but the connection between variations in dopamine levels and the human experience of time has not been directly assessed. Here, we report how dopamine levels in human striatum, measured with sub-second temporal resolution during awake deep brain stimulation surgery, relate to participants’ perceptual judgements of time intervals. Fast, phasic, dopaminergic signals were associated with underestimation of temporal intervals, whereas slower, tonic, decreases in dopamine were associated with poorer temporal precision. Our findings suggest a delicate and complex role for the dynamics and tone of dopaminergic signals in the conscious experience of time in humans.

## One-Sentence Summary

Tonic and phasic dopamine fluctuations in striatum differentially relate to variations in human time perception.

The decisions we make, the memories we retain, and the way we perceive our surroundings are all intrinsically tied to our experience of time (1–4). Therefore, investigations into human cognition and consciousness require consideration of the mechanisms governing time perception, particularly in a time frame on the order of milliseconds to seconds which is integral to basic perceptual and cognitive processes (5–8).

The neurobiological mechanisms underlying the timing of these relatively short intervals remain elusive. Past evidence implicates the striatum and striatal dopamine in time perception (9–17), with higher dopamine levels associated with a tendency to perceive short intervals of time as longer (18–22). This association was drawn largely on the basis of pharmacological experiments and work in non-human animal models (9–13), owing to the inherent challenges of collecting direct dopamine measurements in the living human brain during conscious behaviour (23, 24). However, recent optogenetic work in rodents has introduced a new perspective by demonstrating that rapid changes in dopamine signals (i.e., bursts in phasic dopaminergic neuron activity) may induce temporal underestimation (25). This finding challenges classic timing models that are based on relatively slow response pharmacological effects (18–22, 26). Notably, the contrast between these lines of evidence isn’t just in their resulting interpretation, but also for the very different timescales by which the dopaminergic system was observed to be exerting its influence.

Here we report the first human fast scan cyclic voltammetry (FSCV) (23, 24) study to directly assess the role of sub-second striatal dopamine signals in human time perception. FSCV during the implantation of deep brain stimulation electrodes for the treatment of Parkinson’s disease (PD) symptoms (27) enables the recording of both tonic and phasic striatal dopamine with 100ms precision during concurrent behavioural assessments (23, 24, 28–33). Tonic dopamine refers to the sustained signaling over the course of minutes whereas fast, phasic, dopamine fluctuations occur rapidly within tens to hundreds of milliseconds (34). The present study tests the hypothesis that interval timing is differentially related to dopaminergic activity at varying timescales (34–36), as observed in non-timing cognitive functions (37). Additionally, our approach allows for simultaneous and colocalized measurements of serotonin (28, 30) to assess the neurochemical specificity of any observed effects.

Seventeen healthy controls from a separate study (38) and six patients undergoing deep brain stimulation (DBS) surgery completed a visual temporal bisection task (39). All participants first learned two anchor intervals (500ms and 1,100ms) before judging whether specific intervals (of varying durations between 500ms and 1,100ms) were closer in duration to the short or long anchor interval. This enabled an assessment of temporal accuracy and precision, defined by the degree of systematic error in time judgements and the sensitivity to interval differences, respectively. Concurrently, utilizing a safe and validated human-adapted FSCV protocol (24, 28, 30–33), we recorded in-vivo electrochemical responses at 10 Hz temporal resolution from patients’ caudate (Fig. S1). To estimate dopamine and serotonin concentrations, we trained elastic net penalised regression models using in-vitro calibration data following prior work (24, 28, 30–33, also see Supplemental Information). Subsequently, we quantified both short-lived transient (i.e., phasic) changes and slowly changing (i.e., tonic, at ~4 min timescale) concentrations to investigate the association between these neurochemical signals and changes in participants’ temporal accuracy and precision across trials.

## Elevated *phasic* dopamine concentrations are associated with temporal underestimation

**Fig. 1:**
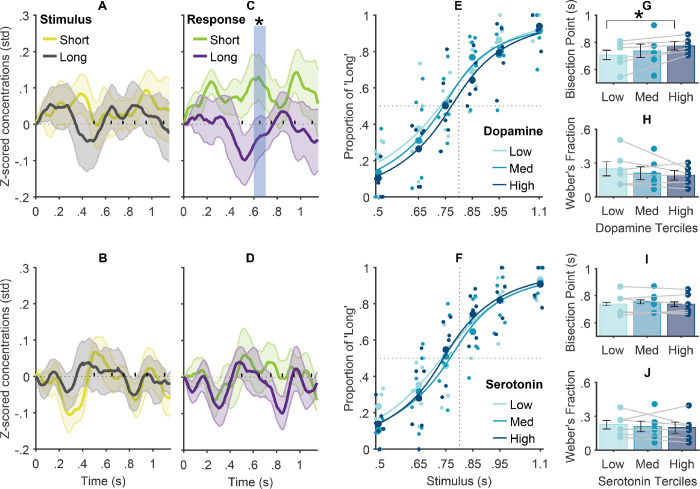
Relationships between phasic caudal changes in dopamine and serotonin levels and stimulus and response parameters. (A-D): Normalized dopamine (A,C) and serotonin (B,D) concentrations from stimulus onset as a function of (A-B) stimulus intervals and (C-D) responses. Shaded areas reflect standard error (SE); x-axis markers on the zero y-axis represent stimulus interval offsets, and the significant cluster*. (E-F) Psychometric functions (PF) fitted to the proportion of ‘long’ responses across stimulus intervals as a function of phasic dopamine (E) and serotonin (F) terciles (Low, Medium, and High). Markers represent individual patient datapoints; dotted lines mark accurate performance with 50% long and short responses (Point of subjective equality [PSE]=0.5) for the true stimulus mid-interval = 0.8 s. (G-J) Indices of the PFs fitted to individual patients’ data. Subjective mid-intervals (Bisection Points) and temporal precision (Weber’s fractions) across dopamine (G-H) and serotonin (I-J) terciles. Markers represent individual patient datapoints. Lines show for each patient the difference in time perception performance as a function of Dopamine or Serotonin tercile. Lower Bisection Point and Weber fraction values denote greater underestimation bias and temporal precision, respectively. * *p*<.050

We tested the hypothesis that elevated striatal dopamine transients are associated with increased temporal underestimation (25). A cluster analysis (40) revealed that short-interval temporal judgements were associated with phasic increases in dopamine levels at 625ms to 670ms after stimulus onset (Fig. 1C), *p*<.050. Notably, this association was not observed when comparing across objectively short and long stimulus durations and no effects were found for serotonin (Fig 1A,B,D, Supplementary Materials). Additional analyses show that the likelihood of short judgements is significantly increased during higher mean single-trial dopamine concentrations, but not serotonin concentrations, in the 625ms to 670ms time window after the stimulus onset (dopamine: *β*=−.11, *p*=.048, serotonin: *β*=−.06, *p*=.28).

To delve further into the relationship between phasic dopamine fluctuations and variations in temporal accuracy and precision, we partitioned trials (*n*_*(total)*_=300) for each patient according to the average dopamine level in the cluster window into low, medium, and high terciles. Using generalized mixed-effects modelling (41), we compared the temporal psychometric functions across these terciles (Fig. 1E–F), where left- and right-ward shifts signify under- and over-estimation biases, respectively, and steeper functions signify superior precision. Consistent with both the foregoing analyses and rodent data (25), our results point to a significant underestimation bias concurrent with higher dopamine transients (Fig. 1E,G; *β*=−.15, *p*=.010). This effect was not observed for serotonin (Fig. 1F,I; *β*=−.01, *p*=.82). Finally, we complemented our analyses with Bayesian assessment of effect prevalence (Fig. S2) which is uniquely suited for experiments with small sample sizes and large trial numbers such as ours (42). The results suggest an 83% probability (with at least 43% at the lower boundary) of detecting statistically significant classification of temporal judgments from dopamine signals within 1,100ms from stimulus onset if our methods are replicated (the lower boundary for prevalence estimate reduces to 33% under more stringent assessment).

Although the observed effects reached statistical significance at 625ms to 670ms from the stimulus onset, our data show that dopamine timeseries for short and long judgments began diverging earlier at around 500ms (short anchor duration) until approximately 800ms (mid-interval of presented stimulus range) (Fig. 1C). This suggests that dopamine neurons may anticipate the impending stimulus offset during this period, which would facilitate efficient decision-making by favouring the selection of the short reference anchor and decreasing the likelihood of classifying the response as ‘long’ when the offset falls within this timeframe. Critically, this plausibly explains why dopamine responses in this time window differed between short and long subjective responses but not following the objective short and long stimulus intervals (Fig. 1A). In line with this interpretation, lower dopamine responses for long judgements might partly reflect temporal discounting of the reward value (43). Notably, in some population clock models, dopaminergic projections modify cortical population dynamics through processes linked to reward prediction error (44), implying that a transient increase in dopamine may lead to temporal underestimation by decelerating trajectories of population dynamics (45, 46). Though, evidence from research in rodents suggests that dopamine neurons exert control over temporal judgments independently of reward processing (25), highlighting the timing specificity of the striatal dopaminergic system as an alternative interpretation. More research is required to further distinguish between these competing hypotheses.

## Elevated *tonic* dopamine concentrations underlie superior temporal precision

**Fig. 2:**
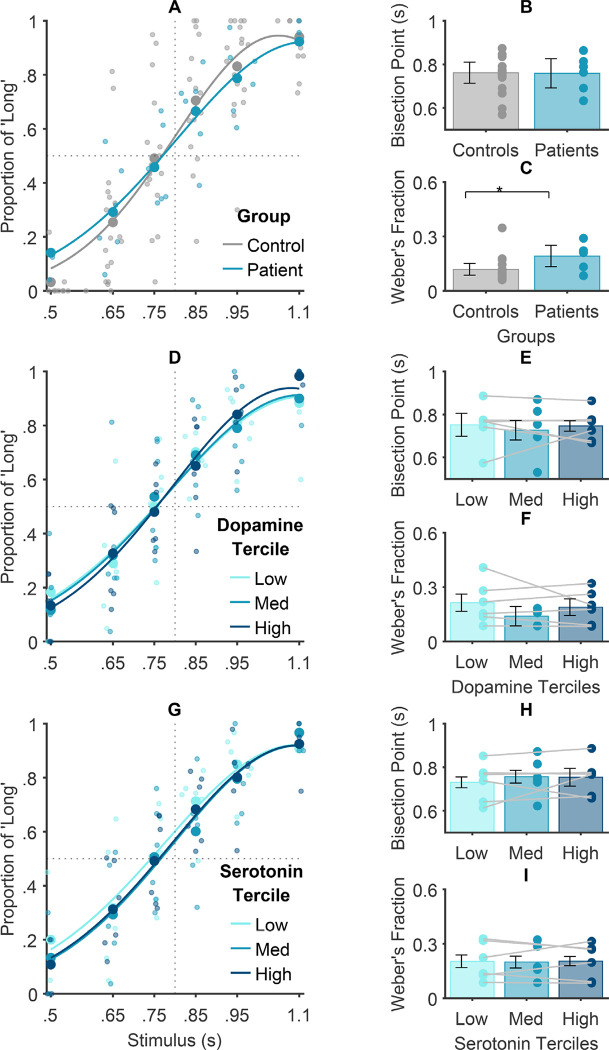
Relationships between tonic caudal changes in dopamine and serotonin levels and time perception performance. (A, D, G) PFs were fitted to the proportion of ‘Long’ responses across stimulus intervals. The horizontal dotted line indicates the point of indifference, i.e., 50% of ‘long’ and 50% of ‘short’ responses. The vertical dotted lines denote the absence of a perceptual bias, such that relative leftward and rightward PF shifts represent the over- and under- estimation biases, respectively. PFs show the fit to (A) patient and control group data, (B) across patients’ dopamine terciles, and (C) patients’ serotonin terciles. (B-C,E-F,H-I) Indices of the PFs fitted to individual patients’ and controls’ data. Subjective mid-intervals (Bisection Points) and temporal precision (Weber’s fractions) across groups (B-C), and patients’ dopamine (E-F) and serotonin (H-I) terciles. Lines show for each patient the difference in time perception performance as a function of Dopamine or Serotonin tercile. Lower Bisection Points (leftward PF shift) and Weber’s fractions (steeper PF slopes) denote increasing underestimation bias and temporal precision, respectively. Markers reflect individual participant datapoints.

Parkinson’s Disease is marked by poorer temporal precision (47–50). Owing to the depletion of dopamine neurons in this condition (51), it has been hypothesized that striatal dopamine concentrations scale with temporal precision. This has been corroborated in pharmacological research (17) but, to our knowledge, has not yet been shown directly with in vivo dopamine measurement in humans. Consistent with this hypothesis, we observed diminished temporal precision in patients (Just Noticeable Difference, JND=.14) compared to controls (JND=.09) (Fig. 2A), *p*=.017, although the Bayesian evidence for this effect was ambiguous, *BF*_*10*_=1.03. Further, we found that patients’ slowly changing tonic dopamine levels over the course of completing the task, predicted variation in their temporal precision (*β*=.50, *p*=.009, *BF*_*10*_=1.70), such that elevated tonic dopamine levels were associated with improved precision (Fig. 2D,F). This effect appeared to be specific to tonic dopamine, as we found evidence supporting the corresponding null hypothesis for serotonin (Fig. 2G,I, *β*=.26, *p*=.35, *BF*_*10*_=.09). Furthermore, exploratory analyses on phasic dopamine and serotonin transients did not suggest a link with temporal precision (Fig. 1E–J; dopamine: *β*=.49, *p*=.39, serotonin: *β*=.61, *p*=.13).

Future research investigating the interplay between across-trial interval learning and tonic dopamine could shed further light on the mechanisms underlying our observed association between tonic dopamine and temporal precision. For example, decreased temporal precision may relate to observed lower motivational states being associated with reduced dopamine levels (52). Our observations may also reflect the role of tonic dopamine in behaviour reinforcement, where a hypoactive tonic firing rate, demonstrated to hinder the extinction of previously reinforced behaviours (53) could contribute to the observed bias towards responses from prior trials in our study. This bias could lead to increased response variability across trials, subsequently diminishing temporal precision.

Our final set of analyses sought to discriminate between two contrasting hypotheses regarding the role of *tonic* striatal dopamine in temporal accuracy. The first hypothesis, grounded in pharmacological research (18–22), predicts a positive association between dopamine and temporal accuracy whereas the second hypothesis predicts no association because of the absence of clear evidence for atypical temporal accuracy in dopamine-depleted PD (54). In alignment with the latter, our observations revealed comparable accuracy across patients and controls (Fig. 2A,B), *p*=.76, with Bayesian evidence for the null hypothesis, *BF*_*10*_=.09. This observation was strengthened by the lack of correspondence between temporal accuracy and variations observed in patients’ tonic dopamine levels (comprising a ~4 min period) over the course of experimental sessions (Fig. 2D,E; *p*=.89, *BF*_*10*_=.06). This was consistent across different analysis window lengths, underscoring that temporal accuracy is not related to steady-state (i.e., slowly changing or tonic) striatal dopamine levels. Similarly, no link was found between temporal accuracy and tonic serotonin fluctuations (Fig. 2G,H; *p*=.24, *BF*_*10*_=.11). These observations challenge internal clock models proposing that higher dopamine levels produce a tendency to perceive time as lasting longer (20). The pharmacological evidence used to support these models (18–22) plausibly reflects mechanisms other than a putative internal clock with its speed of ticking purportedly controlled by striatal dopamine levels. Perhaps most compellingly, our observations reveal differential effects of phasic and tonic dopamine dynamics on temporal accuracy. This suggests a far more intricate relationship between the striatal dopamine system and time perception that warrants further exploration and refinement of existing theoretical frameworks.

Collectively, our observations indicate that changes in tonic dopamine levels may specifically underlie the precision of temporal judgments, whereas we did not observe compelling evidence linking temporal precision to phasic dopamine nor to phasic or tonic serotonin. Coupled with our finding showing that phasic dopamine increases were selectively associated with a higher underestimation bias, these results cumulatively suggest differential roles for tonic and phasic dopamine in human time perception.

## New insights into the role of dopamine in time perception

The present results offer a nuanced perspective on how striatal dopamine fluctuations underlie different features of human time perception. Grace (36) has proposed that dopamine dynamics operate across multiple timescales, encompassing a fast phasic release in response to stimuli and a slow tonic release, which sustains steady-state concentration levels and modulates phasic firing. In line with this perspective, other studies have shown that striatal tonic and phasic firing can yield diverse and even opposing effects on behaviour (37, 55). Investigations into the role of striatal dopamine in time perception faces similar challenges due to conflicting observations linking elevated dopamine with both temporal overestimation (18–22) and underestimation (25, 56). Here we leveraged advances in neurochemical measurement methods in human participants to show that heightened phasic bursts of striatal dopamine are uniquely associated with temporal underestimation whereas higher tonic dopamine concentrations correlated with superior temporal precision. Moreover, our results highlight the neurochemical specificity of these effects as our analyses suggest that the observed effects are specific to dopamine fluctuations and are not observed with corresponding variations in serotonin levels, which have been linked to interval timing in pharmacological studies (57–59).

Dopamine assumes a central role in brain function regulation and is implicated in a broad spectrum of disorders, including Parkinson’s disease (51). These roles, rooted in dopamine’s evolutionary antiquity, persist across species (60). Recent shifts in dopamine transmission models have recognized the importance of spatiotemporal precision in certain dopamine functions. A specialized architecture has been proposed, involving release-receptor assemblies at micrometre scales, to account for the distinct functions associated with the tonic concentrations and transient bursts of activity of dopaminergic neurons (34). This theoretical framework emphasizes a more refined spatiotemporal resolution of dopamine signalling to mediate diversity of functions within the striatal dopamine system. Ongoing research investigating the principles of these theories may profoundly impact our understanding of past evidence linking a wide range of phenomena to the function of the striatal dopamine system, including time perception.

Studies employing FSCV in animal models have shed light on how dopaminergic transmission and dopamine dynamics contribute to the neurochemical mechanisms of drug addiction, PD, and schizophrenia (61). Nonetheless, until recently, such research had not been feasible in the human brain, owing to various methodological hurdles (23, 24, 62). Our results suggest new directions for research that harness recent methodological advances in the measurement of neurochemical dynamics (33, 63) and their function in time perception and related behaviour in humans. Advancing our understanding of the neurobiological foundations of human time perception will refine existing theories (20, 44, 64, 65), impacting healthy and dysfunctional timing and the knowledge concerning the timing in human cognition more broadly. This includes implications for metacognition, reinforcement learning, reward processing, and understanding neurological and psychiatric conditions like schizophrenia, addiction disorders and impulse control that involve aberrant dopamine functioning (66, 67). For example, recent research indicates that heterogeneity in PD symptoms may be accounted for by differences in time perception, with implications for understanding dopaminergic mechanisms in time perception and PD symptomatology (38). These insights are crucial for developing nuanced treatment approaches and enhancing our comprehension of the complex interplay between dopamine, time perception, and human cognition. Consequently, human FSCV emerges as a valuable set of methods for investigating the neurochemical underpinnings of human time perception and germane cognitive functions.

## Supplementary Material

Supplement 1

## Data Availability

All data and code will be released with a 1-year embargo from the publication date.
