# Supplementary material for: Sub-second and multi-second dopamine dynamics underlie variability in human time perception
Source: medRxiv. 2024 Feb 9:2024.02.09.24302276. Preprint. [Version 1] doi: 10.1101/2024.02.09.24302276 (PMC10871373; doi:10.1101/2024.02.09.24302276)
Supplement: Supplement 1 [file NIHPP2024.02.09.24302276v1-supplement-1.pdf]

medRxiv preprint doi: https://doi.org/10.1101/2024.02.09.24302276; this version posted February 9, 2024. The copyright holder for this preprint (which was not certified by peer review) is the author/funder, who has granted medRxiv a license to display the preprint in perpetuity.
It is made available under a CC-BY 4.0 International license .

Supplementary Materials for

Sub-second and multi-second dopamine dynamics underlie variability in human time perception

Renata Sadibolova, Emily DiMarco, Angela Jiang, Benjamin Maas, Stephen B. Tatter, Adrian Laxton, Kenneth T. Kishida, & Devin B. Terhune

Corresponding authors: renata.sadibolova@roehampton.ac.uk, kkishida@wakehealth.edu, devin.terhune@kcl.ac.uk

Materials and Methods

The study comprised two sessions (1 to 10 days apart for the patients and the same day with a ten-minute break between sessions for controls), each including training and experimental phases. All participants achieved 65% accuracy in 20 consecutive training trials, with a single patient and a single control requiring, respectively, 48 and 30 trials in the first session and another patient requiring 21 trials in the second session. Control participants and patients not in the surgical setting (session 1) were seated at a desk approximately 70 cm from the monitor and completed four blocks of fifty experimental trials. During surgery (session 2), PD patients sat in a semi-upright position and viewed the monitor at a distance of approximately 100 cm. Patients were on their dopamine replacement medication in session 1 whereas their medication was withheld in session 2 at least 12 hours prior to their surgery (*27*). Our experimental protocol afforded up to 30 minutes for the experimental task with concurrent FSCV recording (*24*, *32*), during which PD patients completed six blocks of 50 experimental trials.

Participants

Six patients with Parkinson's disease (PD) (2 females, 4 males) between the ages of 62 and 73 years (M_{Age}=67.7, *SD*=4.5) participated in this study during DBS surgery. For behavioural analyses only, we additionally included a sample of 17 healthy controls (7 females, 10 males, aged 50-66; M_{Age}=57.5, *SD*=5.0) from a prior study (*38*), with no psychiatric or neurological conditions. We excluded 3 patients with a psychiatric diagnosis. All participants provided informed written consent in accordance with approval by the IRB committee at Wake Forest University Health Sciences (IRB00017138 and IRB00044216).

Experimental task

Participants completed a visual temporal bisection task with stimulus presentation implemented with Psychtoolbox-3 (*68*) in MATLAB R2018b (MathWorks, Natick, USA). Each trial consisted of a jittered interstimulus interval (ISI1; blank black screen) drawn from a truncated Poisson distribution (400-600ms), a white circle (~2° of visual field) that varied in duration (500, 650, 750, 850, 950, or 1100ms), a second interstimulus interval (ISI2; blank black screen; 900ms), and a two-alternative forced choice judgment prompt ("S"=short; "L"=long). The response prompt on screen (i.e., [S L] or [L S]) was randomized on every

medRxiv preprint doi: https://doi.org/10.1101/2024.02.09.24302276; this version posted February 9, 2024. The copyright holder for this preprint (which was not certified by peer review) is the author/funder, who has granted medRxiv a license to display the preprint in perpetuity.
It is made available under a CC-BY 4.0 International license .

trial to counterbalance handedness and visual presentation effects. Participants responded with the left or right shoulder keys of a Logitech game controller (Logitech International S.A.), corresponding to the respective response letter location on the monitor. In the 20-trial training phase, participants learnt two anchor intervals (500 and 1100ms) comprising equal proportions of each stimulus. Additional training trials automatically followed if accuracy was below 65% until this target was reached in the lattermost 20 trials or until the maximum training time of 7 minutes had passed. In the subsequent experimental phase, participants were presented with white circles of varying intervals (500-1100ms) and judged whether they were closer in duration to the trained short or long anchor intervals. All six experimental (50-trial) blocks began with two reminders of each anchor stimulus (4 trials); the remaining 46 trials included 10 repetitions of the 4 middle stimuli and 3 repetitions of each anchor stimulus.

Fast-Scan Cyclic Voltammetry (FSCV)

Fast scan cyclic voltammetry (FSCV) was performed using carbon fibre microelectrodes surgically placed in the caudate (Fig. S1; 6 channels, 5 patients) or thalamus (Fig. S1; 1 channel, 1 patient, excluded to focus analyses on caudate region). Briefly, the FSCV measurement protocol is the same as previously reported in humans: hold working electrode at -0.6V for 90ms, ramp to +1.4 V and back to -0.6V at 400 V/s, and repeat, for an overall electrochemical sampling rate of 10 Hz frequency reported (*23*, *24*, *63*, *28–33*, *38*, *62*). The resulting raw electrochemical current was measured at a sampling rate of 250kHz, thus providing a raw 10ms voltammogram (2500 samples at 250KHz). These data were subjected to analysis as described previously (*24*, *28*) and below.

medRxiv preprint doi: https://doi.org/10.1101/2024.02.09.24302276; this version posted February 9, 2024. The copyright holder for this preprint (which was not certified by peer review) is the author/funder, who has granted medRxiv a license to display the preprint in perpetuity.
It is made available under a CC-BY 4.0 International license .

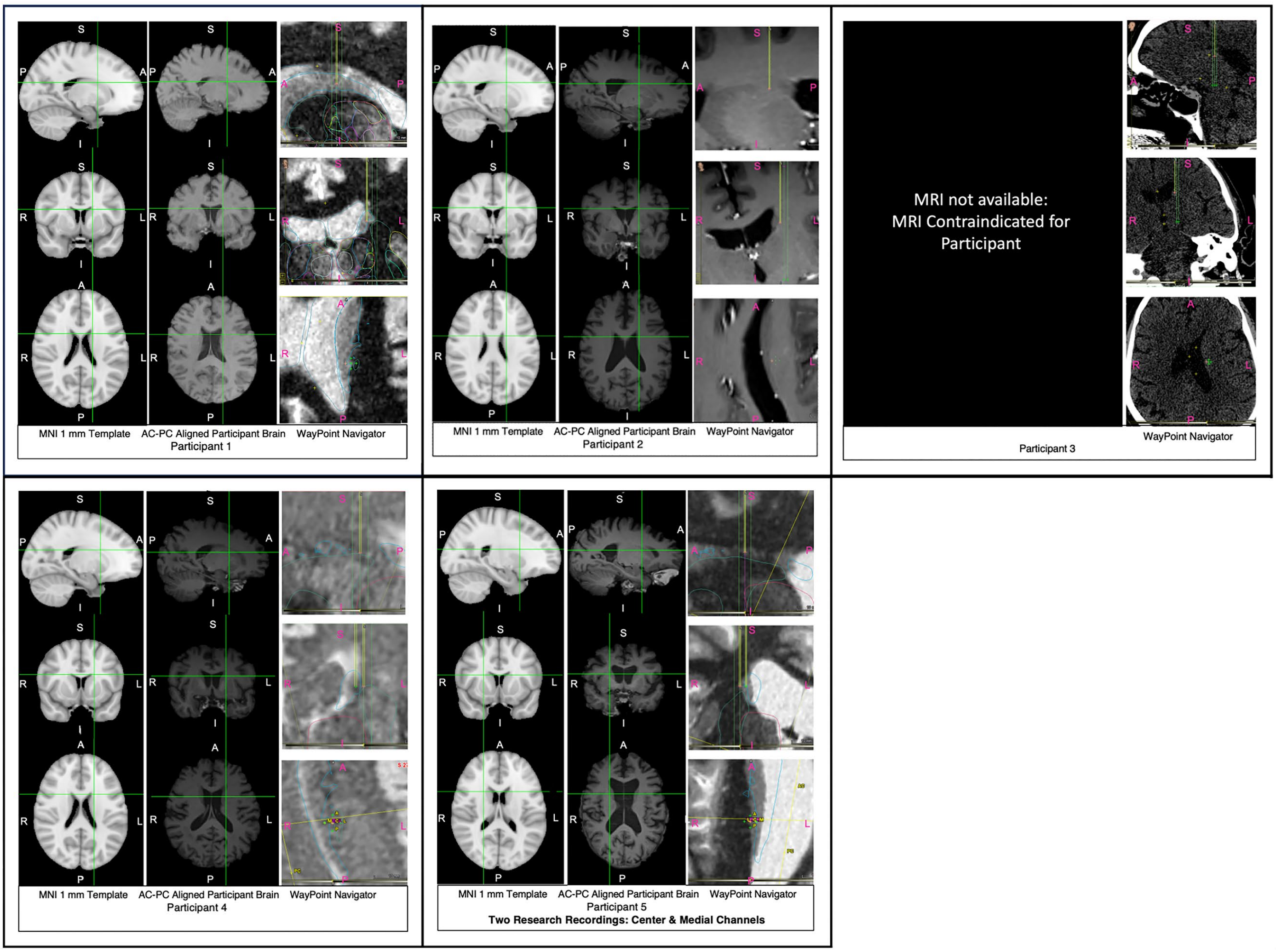

Figure S1. Electrode Coordinate Locations on MNI 152 Template, AC-PC aligned, and WayPoint Navigator Software. For each participant, from left to right, electrode coordinates are depicted on the MNI 152 1 mm template, AC-PC aligned participant brains, and WayPoint Navigator Surgical Planning Software Suite. Normalization to the MNI 152 template was achieved through FSL(*69*). Each figure panel shows sagittal, coronal, and axial planes for each participant from top to bottom. An exception is noted for Participant 3, for whom MRI was contraindicated and unavailable. For Participant 5, two distinct recordings were obtained from the same depth, one from a center channel, and another from a medial channel with a 2 mm offset. Abbreviations: AC-PC = anterior commissure-posterior commissure; S = superior; I = inferior; P = posterior; A = anterior; R = right; L = left; MRI = magnetic resonance imaging.

medRxiv preprint doi: https://doi.org/10.1101/2024.02.09.24302276; this version posted February 9, 2024. The copyright holder for this preprint (which was not certified by peer review) is the author/funder, who has granted medRxiv a license to display the preprint in perpetuity.
It is made available under a CC-BY 4.0 International license .

Analyses

Behavioural data analysis

Trials with premature responses (during ISI2) were excluded (*M*=1.50, *SD*=1.76, range: 0-5). We first tested whether psychophysical markers of time perception differed between controls and PD patients. Using the *MixedPsy* package (*41*) in R (*70*), we fitted a generalized linear mixed-effects model (GLMM) to the proportion of long responses, including the group and demeaned stimulus intervals and their interaction as fixed-effects parameters and stimulus interval slopes and intercepts for participants as random effects. Temporal accuracy (Point of Subjective Equality; PSE or Bisection Point; BP) and temporal precision (Just Noticeable Difference; JND, or Weber's Fraction; WF) were derived, respectively, from the intercepts and slopes of a cumulative normal distribution (probit) function (*41*). Leftward (negative PSE) and rightward (positive PSE) shifts of this function reflect, respectively, temporal over- and under- estimation biases whereas a steeper function (lower JND) reflects greater temporal precision.

Neurochemical concentrations

We trained statistical models for optimal out-of-probe prediction of striatal DA and SE concentrations following prior work (*24*, *28*, *30*) and applied these models to estimate dopamine and serotonin concentrations from our human FSCV data. To this end, we produced the calibration data with known concentration labels following an in-vitro FSCV protocol described in detail elsewhere (*24*, *28*, *30*). Calibration data were collected using the same protocol as that used in the human experiments. Calibration data consisted of 10ms voltammograms recorded every 100ms (each voltammogram = 2500 samples at 250kHz), thus yielding 10Hz overall temporal resolution of DA and SE signal time series. We first sought to specify the set of parameters for optimal machine learning performance on naïve probes not included in the training. This was achieved by down-sampling calibration voltammograms by averaging the bins of every 30 samples for dopamine and 10 samples for serotonin. Each down-sampled voltammogram was concatenated with its first and second derivative with the resulting 249 (dopamine) and 747 (serotonin) samples submitted as predictors to an elastic-net regularized linear regression model (*71*, *72*). The outcome variable was a multivariate Gaussian distribution consisting of known concentration labels for pH, 3,4-dihydroxyphenylacetic acid (DOPAC), L-ascorbic acid (LAA) and dopamine (DA) or serotonin (SE). We trained separate models for DA and SE estimation using a 10-fold cross-validation procedure whilst searching for minimal mean squared error (unexplained variance) in a 2D penalty parameter space (penalty weight λ and a mixing term α, see Qian et al., 2013). The λ values were determined by an inbuilt function (*71*) and the α range was 0 to 1 in steps of 0.25. Our approach allowed for the reliable and generalized estimation of DA and SE concentrations against a background of varying pH activity as shown by model performance on two naïve calibration probes withheld from model-training (Root Mean Squared Error: dopamine = 0.632, serotonin = 0.113; Signal to Noise Ratio: dopamine = 3.99, serotonin = 18.91).

Phasic signal change and time perception

The final model estimates for patients' in-vivo DA and SE concentrations were up-sampled by linear interpolation to 1000 Hz for the purposes of epoching. We extracted epochs relative to stimulus onset or stimulus offset and z-scored the signal (±3 sec relative to stimulus onset) to subtract away fluctuations at slower timescales. Insofar as the z-score

medRxiv preprint doi: https://doi.org/10.1101/2024.02.09.24302276; this version posted February 9, 2024. The copyright holder for this preprint (which was not certified by peer review) is the author/funder, who has granted medRxiv a license to display the preprint in perpetuity.
It is made available under a CC-BY 4.0 International license .

normalization brings the epochs into a common frame of reference without affecting their individual shapes, we compared the transient within-trial signal changes both within and across participants.

Our next objective was to assess if patients' binary response patterns in the temporal bisection task varied with transient changes in caudal DA and SE signals during and after stimulus presentation. We analysed timeseries aligned to stimulus onset [0 to 1400ms] and offset [-500 to 900ms]. We applied a cluster mass test procedure (*73*, *74*) for detecting the time window where neurochemical concentrations differed at the group level as a function of the stimulus interval, response (short or long), and their interaction, including the by-participant random error for the interaction term in the model. Clusters were identified with the threshold-free cluster enhancement (*tfce*) method (*75*) and assessed for significance with the *Rde_keradPajouh_renaud* (*76*) permutation method (10,000 iterations) in the *permuco* package (*40*) in R (*70*).

In the subsequent analyses, we submitted mean single-trial concentrations in identified cluster window(s) and stimulus interval (short vs. long) as fixed-effects predictors in a GLMM with binary responses (short vs. long) as the outcome variable and patient probes as the random intercept term. We additionally demarcated trials for individual probes into low, medium, and high DA and SE terciles of the mean signal distribution in the cluster window(s) and fitted GLMMs with signal tercile and stimulus predictors, proportion of long responses as the outcome variable, including the uncorrelated random intercepts and by-stimulus slopes for the probe. This allowed us to assess whether transient neurochemical fluctuations related to psychophysical indices of temporal accuracy and precision (*41*).

Although appropriate for its improved statistical power in dealing with multiple comparison tests (*40*, *73*), the cluster permutation analysis disregards random effects associated with participants and their interactions with fixed effects. Whereas the GLMM remedies some of those issues, we complemented these analyses with Bayesian assessments of the effect prevalence, which shifts the focus from population mean estimates to individuals and is particularly well-suited for experiments with small sample sizes and large trial numbers (*42*). For each PD patient, we trained 10-fold cross-validated logistic regression models in a sliding window across the signal time series (200ms width, steps of 20ms) with neurochemical concentrations as predictors and binary responses as the outcome. To ensure that the models were unbiased, the predictors were normalized, and the training data were balanced by subsampling trials from the response class with more trials, so that the number of trials was matched to the class with fewer trials. The minimal size of a training dataset in this study was N=212 trials. Area under the curve (AUC) was computed to assess classification performance with values of 0.50 and 1.00 representing chance and perfect classification, respectively. We applied one-tailed one-sample t-tests to compare AUC values (10 folds) against 50% chance performance at each timepoint for each participant. The alpha level was lowered to 0.01 to correct for multiple comparisons (46 time points); however, given that this is the first human study investigating in-vivo human sub-second neurochemical fluctuations relating to time perception, we additionally include the results without the multiple comparison correction (*28*). The Bayesian prevalence analysis provides the population prevalence estimate (maximum a posteriori [MAP]) together with its associated uncertainty intervals (highest posterior density intervals [HPDI] or lower bound posterior quantiles). Expanding upon the traditional prevalence analysis, we also followed Ince et al.'s (*42*) approach to obtain prevalence estimates for different effect sizes.

Tonic signal change and time perception

medRxiv preprint doi: https://doi.org/10.1101/2024.02.09.24302276; this version posted February 9, 2024. The copyright holder for this preprint (which was not certified by peer review) is the author/funder, who has granted medRxiv a license to display the preprint in perpetuity.
It is made available under a CC-BY 4.0 International license .

Our previous analyses were complemented with further mixed-effects modelling to assess the role of tonic DA concentrations in time perception. To facilitate comparison across all patient probes, we z-scored the concentrations observed for ongoing stimuli using the mean and standard deviation of these concentrations across all trials. We subsequently computed baseline-corrected mean concentrations for individual trials and probes. These were then averaged and the proportions of "long" responses for each stimulus interval were computed for each window of 100 trials moving forward in ~60-second (~25-trial) increments. The robustness of this analysis was further assessed across different window lengths: 50, 75, 125 and 150 trials (Supplemental materials). A GLMM (probit link) was then fitted to the proportion of long responses, including tonic DA concentrations and demeaned stimulus intervals and their interaction as fixed-effects parameters and by-stimulus slopes and intercepts for patient probes as random effects. As described above, this method yielded psychometric functions with separate beta coefficients reflecting temporal accuracy and precision (*41*). The windows were additionally partitioned according to the average DA concentrations into low, medium, and high DA terciles, thereby enhancing our ability to visualize the results. Finally, all analyses were repeated with SE concentrations to assess the neurochemical specificity of our findings. The Bayesian prevalence estimate was not computed for this analysis due to the small number of trial-windows.

Supplementary Text

Time perception in PD

We assessed two contrasting hypotheses for the role of tonic striatal DA in temporal accuracy. The first, grounded in prior pharmacological studies (*20*, *77*, *78*), predicted a positive relationship, and the second, given the absence of clear evidence in dopamine-depleted PD (*54*), predicted no association. We observed a trend for a leftward shift of a psychometric function denoting an overestimation trend (Fig. 2A) in both PD patients, PSE_{PD}=-.03 s, SE=.03, [95% CI:-.08, .02], and controls, PSE_{CTRL}=-.04 s, SE=.03, 95% CI [-.08, .01], which, in line with the second hypothesis, was similar across the groups, β=.10, SE=.33, 95% CI [-.58, .79]), z=.31, p=.76, with Bayesian evidence for the null hypothesis, BF_{10} = .09.

We additionally hypothesised that lower tonic DA levels will correspond with poorer precision (*49*, *50*). As expected, temporal precision was poorer in PD patients, JND_{PD}=.14, SE=.02, 95% CI [.08, .19], as indicated by higher values (i.e., flatter slopes; Fig. 2A), than in controls, JND_{CTRL}=.09, SE=.01, 95% CI [.08, .10], β=2.44, SE=1.03, 95% CI [.33, 4.56]), z=2.37, p=.02, BF_{10} = 1.03. These results underscore diminished temporal precision in PD patients, corroborating previous findings amid some contrasting evidence (*49*, *50*).

Caudal dopamine and serotonin transients and time perception

We next tested the prediction that the elevated striatal DA transients are linked to temporal underestimation, as suggested by animal research (*25*). Toward this end, we investigated how patients' neurochemical signals tracked variations in their temporal responses. Lower caudal DA after stimulus onset [625 to 670 ms] was associated with increased tendency to judge stimulus intervals as long, cluster p_{TFCE} <0.05 (Fig. 1C). This effect was dopamine-specific with no clusters found in SE time series (Fig. 1D). Importantly, the cluster analyses did not yield any statistically significant differences in DA or SE

medRxiv preprint doi: https://doi.org/10.1101/2024.02.09.24302276; this version posted February 9, 2024. The copyright holder for this preprint (which was not certified by peer review) is the author/funder, who has granted medRxiv a license to display the preprint in perpetuity.
It is made available under a CC-BY 4.0 International license .

concentrations as a function of actual stimulus interval (modelled as both a binary variable [Fig. 1A,B] and with six stimulus levels). Finally, no clusters were identified in signal timeseries aligned to stimulus offset. Cumulatively, these results suggest that short responses are associated with larger bursts of DA activity during stimulus processing.

A complementary GLMM analysis corroborated that lower phasic DA at 625 – 670ms from stimulus onset was associated with increased long responses, β=-.11 (95% CI [-.22, -.001]), *SE*=.06, *t*=-1.97, *p*=.048, whereas SE was not a significant predictor of temporal estimates in this time window, β=-.06 (95% CI [-.16, .05]), *SE*=.05, *t*=-1.07, *p*=.28. This effect is further reflected in our analysis of a psychophysical measure of temporal accuracy (PSE): lower DA was associated with a greater overestimation bias, β=-.15 (95% CI [-.27, -.04]), *SE*=.06, *z*=-2.58, *p*=.01 whereas this effect was not significant for serotonin, β=-.01 (95% CI [-.13, .10]), *SE*=.06, *z*=-.22, *p*=.82. By contrast, temporal precision (JND) did not differ across phasic DA terciles, β=.34 (95% CI [-.43, 1.13]), *SE*=.40, *z*=.86, *p*=.39, or SE terciles, β=.61 (95% CI [-.18, 1.42]), *SE*=.41, *z*=1.51, *p*=.13 (Fig. 1E-J). These results complement the previous analysis and demonstrate that intra-individual variability in timing accuracy is related to transient caudal DA fluctuations, with evidence for neurochemical specificity.

To gain a deeper insight into these effects, we used a Bayesian approach to estimate population prevalence. This approach offers several benefits compared to traditional population mean hypothesis testing, including the capability to infer population-level estimates and their precision in studies with small participant numbers and to derive an estimate irrespective of the specific window where classification may peak in different participants (*42*). The AUC for classification of short and long responses from DA concentrations within individual participants varied from .50 to .59 (59% classification accuracy), reflecting heterogeneity across patients (Fig. S2 top-left). For example, whereas patient 20's AUC ranged from .50 to .53, reflecting poor classification, patient 19 displayed the highest AUC values (.55<AUC<.60) around anchor stimulus interval offsets (500 and 1100 ms). The Bayesian estimate of population prevalence based on classification performance peaked at 47.38% at 620ms, which corresponds with our earlier results (Fig. 1). The Bayesian maximum a posteriori (MAP) (*79*) estimate of the effect prevalence was 82.50% (96% highest posterior density interval [HPDI]: [42.50, 98.80]) with uncorrected within-subject threshold α=.05 (Fig. S2 top-right). This suggests that there is at least a 42.50% chance of observing statistically significant classification of temporal judgments from DA signals within 1100ms from stimulus onset in a new sample if our methods are replicated (the prevalence estimate reduces to 32.70% with a more stringent threshold α=.01; Fig. S2 bottom-right).

medRxiv preprint doi: https://doi.org/10.1101/2024.02.09.24302276; this version posted February 9, 2024. The copyright holder for this preprint (which was not certified by peer review) is the author/funder, who has granted medRxiv a license to display the preprint in perpetuity.
It is made available under a CC-BY 4.0 International license .

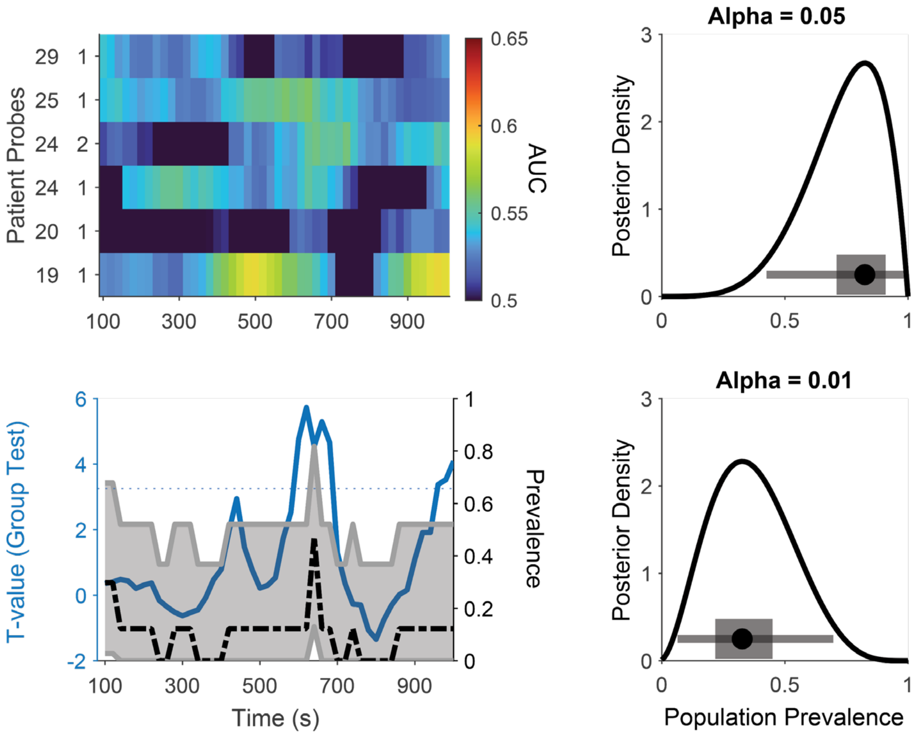

Figure S2. Bayesian population prevalence for classification of short and long responses from dopamine timeseries. (**Top-left**): Heatmap of AUC values for each patient probe (rows) and time window (200ms width, centred on x-axis labels). The blue solid line in **bottom-left** panel tracks the group-level t-test values, and the dotted blue line denote the threshold at α=0.01. The posterior distribution of population prevalence is shown for an effect in the analysed time series (cumulative density function in **right panels**) and at each timepoint (dashed black line in **bottom-left panel**). (**Right panels**) The grey horizontal line represents 96% highest posterior density intervals (HPDI), and the short rectangle shows the 50% HPDI.

Tonic caudal dopamine and serotonin concentrations and time perception

Our next set of analyses evaluated the association between within-subject variations in interval timing and *tonic* caudal DA levels. We contrasted the competing predictions: one suggesting that tonic DA is unrelated to temporal accuracy, given inconclusive evidence for atypical temporal bias in PD (*54*), and the other proposing a positive correlation, supported by pharmacological evidence (*22*, *80–82*). Consistent with the first hypothesis and our behavioural observations, tonic DA was not associated with temporal bias, β=-.004 (95% CI [-.06, .05]), *SE*=.03, *z*=-.14, *p*=.89, BF_{10} = .06, underscoring that temporal accuracy is not related to steady-state (tonic) caudal DA levels (Fig. 2D,E). This finding stands in stark contrast to the compelling link observed for phasic DA levels. Furthermore, we also observed no association between tonic SE and temporal bias, β=-.04 (95% CI [-.12, .03]), *SE*=.04, *z*=-1.17, *p*=.24, BF_{10} = .11 (Fig. 2G,H).

We additionally predicted that lower tonic DA levels, prevalent in PD, will correspond with poorer precision (*49*, *50*). Our behavioural data analysis supported this hypothesis, revealing poorer precision for patients than controls. Crucially, in our patient sample, temporal precision showed a positive association with their tonic DA concentrations (as shown by steeper slopes for high DA terciles in Fig. 2D), β=.50 (95% CI [.13, .87]), *SE*=.19,

medRxiv preprint doi: https://doi.org/10.1101/2024.02.09.24302276; this version posted February 9, 2024. The copyright holder for this preprint (which was not certified by peer review) is the author/funder, who has granted medRxiv a license to display the preprint in perpetuity.
It is made available under a CC-BY 4.0 International license .

z=2.61, p=.01, BF_{10} = 1.70, although the Bayesian evidence was not conclusive. We did not find a similar association with serotonin levels (Fig 2G), β=.26 (95% CI [-.28, .80]), SE=.27, z=.94, p=.35, BF_{10} = .09. These findings support the hypothesis that fluctuations in tonic DA, but not SE, are linked to temporal precision. As observed earlier for temporal accuracy, the results for temporal precision further emphasize the contrast in the impact of tonic vs. phasic dopamine concentrations on interval perception, highlighting potentially differential effects of distinct temporal dynamics of DA activity.
